# Population and antenatal-based HIV prevalence estimates in a high contracepting female population in rural South Africa

**DOI:** 10.1186/1471-2458-7-160

**Published:** 2007-07-18

**Authors:** Brian D Rice, Jörg Bätzing-Feigenbaum, Victoria Hosegood, Frank Tanser, Caterina Hill, Till Barnighausen, Kobus Herbst, Tanya Welz, Marie-Louise Newell

**Affiliations:** 1 Africa Centre for Health & Population Studies, KwaZulu Natal, South Africa; 2 Department for Infectious Disease Epidemiology, Robert Koch-Institute, Berlin, Germany; 3 Centre for Population Studies, London School of Hygiene and Tropical Medicine, London, UK; 4 Department of HIV and Genitourinary Medicine, King's College London School of Medicine at Guy's, King's College and St Thomas' Hospitals, London, UK; 5 Centre for Paediatric Epidemiology and Biostatistics, Institute of Child Health, UCL, London, UK

## Abstract

**Background:**

To present and compare population-based and antenatal-care (ANC) sentinel surveillance HIV prevalence estimates among women in a rural South African population where both provision of ANC services and family planning is prevalent and fertility is declining. With a need, in such settings, to understand how to appropriately adjust ANC sentinel surveillance estimates to represent HIV prevalence in general populations, and with evidence of possible biases inherent to both surveillance systems, we explore differences between the two systems. There is particular emphasis on unrepresentative selection of ANC clinics and unrepresentative testing in the population.

**Methods:**

HIV sero-prevalence amongst blood samples collected from women consenting to test during the 2005 annual longitudinal population-based serological survey was compared to anonymous unlinked HIV sero-prevalence amongst women attending antenatal care (ANC) first visits in six clinics (January to May 2005). Both surveillance systems were conducted as part of the Africa Centre Demographic Information System.

**Results:**

Population-based HIV prevalence estimates for all women (25.2%) and pregnant women (23.7%) were significantly lower than that for ANC attendees (37.7%). A large proportion of women attending urban or peri-urban clinics would be predicted to be resident within rural areas. Although overall estimates remained significantly different, presenting and standardising estimates by age and location (clinic for ANC-based estimates and individual-residence for population-based estimates) made some group-specific estimates from the two surveillance systems more predictive of one another.

**Conclusion:**

It is likely that where ANC coverage and contraceptive use is widespread and fertility is low, population-based surveillance under-estimates HIV prevalence due to unrepresentative testing by age, residence and also probably by HIV status, and that ANC sentinel surveillance over-estimates prevalence due to selection bias in terms of age of sexual debut and contraceptive use. The results presented highlight the importance of accounting for unrepresentative testing, particularly by individual residence and age, through system design and statistical analyses.

## Background

In sub-Saharan Africa, surveillance of women attending antenatal care (ANC) is often used to measure prevalence and monitor trends in HIV infection. However, when applying ANC-based HIV prevalence estimates to the general population the following biases should be considered: only pregnant women are eligible for testing (structural bias) [1]; women who become pregnant and attend ANC facilities are sexually active and not using contraceptives (self selection bias) [1,2]; attendance varies by factors associated with HIV [2]; and, HIV-infected women may be less likely to become pregnant [1-7].

Fertility among HIV-infected women in sub-Saharan Africa is lower than in HIV-uninfected women, except in women aged 15–19 years [7]. In young women the selective pressure of sexual debut on pregnancy and HIV infection resulted in higher fertility rates among the HIV infected [7]. Six studies conducted in high fertility populations in sub-Saharan Africa showed that HIV prevalence in pregnant women was lower than in women of reproductive age overall [6]. In populations of southern Africa with low fertility and extensive contraceptive use, bias due to the selection for pregnancy in ANC-based HIV prevalence estimates could be smaller [8]. This is because women who use modern methods of family planning may take more effective measures to avoid HIV infection [1], and sub-fertility in HIV-infected women will have a weaker effect [8].

Bias due to the purposive selection of ANC facilities should also be considered when applying ANC-based prevalence estimates to a population [1,8]. Over-representation of ANC clinics in urban areas, where HIV prevalence is usually relatively high, may result in HIV prevalence levels being exaggerated [8]. However, evidence of urban and peri-urban based clinics attracting large numbers of women from rural areas, where HIV prevalence tends to be lower, would mitigate this [8].

Population-based surveys are more representative of the general population than ANC-based surveys as they include non-pregnant and non-ANC attending pregnant women, as well as men. However, limitations exist and of particular concern is the effect of non-response on HIV prevalence estimates.

Population-based surveys have been conducted in several sub-Saharan African countries [8-20], including South Africa where primary health care services are free, use of modern contraception is high and the total fertility rate is low [21]. It has been suggested that women using modern methods of family planning may take more effective measures to avoid HIV infection [1]. In KwaZulu Natal, South Africa, information collected by the Africa Centre Demographic Information System (ACDIS) [21-23], in 2001 shows 51.7% of women aged 15 to 49 years to have ever used a modern contraceptive method, the median age of first sexual intercourse to be 17.7 years, and fertility between 1980/84 and 2000/01 to have declined in women aged 18 years and older [21].

In this paper we present and compare HIV prevalence estimates from both population-based surveillance and ANC sentinel surveillance within an area of sub-Saharan Africa with high contraceptive use and low fertility. We identify and explore differences, and reasons for differences, between the two surveillance systems, with particular emphasis on unrepresentative selection of clinics in ANC sentinel surveillance and unrepresentative testing in population-based surveillance. Where estimates from the two surveillance systems differ we explore methods to reduce these differences.

## Methods

The ACDIS is conducted in the rural sub-district of Hlabisa in northern KwaZulu-Natal, South Africa. It covers 435 square kilometres and a total resident population of 85123 (unpublished data as of January 2005). The 11284 homesteads within the area have been enumerated and mapped using a geographic information system (GIS). The area includes a formally designated urban township, peri-urban areas (settlements with a population density of more than 400 people per km^2^), and rural areas. The rural population live in scattered homesteads that are not concentrated in villages.

Population-based linked anonymous HIV testing was introduced within the ACDIS in July 2003. Sampling for testing is based upon information collected routinely through demographic surveillance [21-23]. All resident women aged 15 to 49 years and men aged 15 to 54 years, were eligible for annual HIV testing through a finger-prick blood sample on filter paper and approached for inclusion in the survey [24-27]. Additionally, 10% of non-resident members of households located within the study area, in above age groups, were randomly selected for testing. To facilitate a comparison with ANC-based estimates, only resident women were included in these analyses. A resident is an individual, reported by the household informant, who keeps their daily belongings, and who spends most nights, within the survey area [23,24]. Results are those from the second annual HIV survey (January to December, 2005).

Ethical approval was received from the University of KwaZulu Natal (E029/2003). All individuals eligible for HIV testing were asked for written informed consent and informed about the potential risks to becoming aware of ones HIV status, about how and where HIV test results and post-test counselling may be accessed and, if found positive, how they may be referred to a local clinic for further screening and assessment of eligibility for antiretroviral treatment. The choice to provide a test sample and to access the HIV test result rests fully with the individual.

In December 2001, Hlabisa Health sub-district became the first rural district in South Africa to provide antiretroviral drugs for the prevention of HIV mother to child transmission. Between January and May 2005, alongside the Prevention of Mother-to-Child Transmission (PMTCT) programme, venous blood was taken for routine ANC laboratory tests from all women attending first ANC visits at all six government clinics delivering ANC within the ACDIS. Surplus blood from these samples was also used for anonymous unlinked HIV testing. Parity and age was linked to a woman's HIV test result. Results cannot be linked back to the individual as, apart from date of birth, no personal identifiers were collected.

For each participant the most likely clinic at which antenatal care was obtained was predicted on the basis of a GIS accessibility model that estimated travel time to the six government clinics within the surveillance area offering ANC. The model took into account the quality and distribution of the road network, barriers to movement and the likelihood of utilising public transport to access care [28]. The six clinics were categorised as mixed peri-urban/urban, mixed rural/peri-urban or rural respectively on the basis of their predicted constituent catchment populations.

As ANC sentinel surveillance does not collect residency information, ANC attendees were proportionally assigned to one of the three residency types (urban/peri-urban/rural) based on the underlying predicted catchment population of the clinic attended. To assess the reliability of the clinic accessibility model in predicting ante-natal attendance we compared the prediction of the model with reported ante-natal clinic usage amongst women ever reporting a pregnancy within the ACDIS.

Pearson chi^2 ^values and all confidence intervals presented are at the 95% level. STATA 9.0 (Stata Corp., College Station, Texas, USA) was used for univariate and multivariate analyses. To account for unrepresentative testing, population-based and ANC-based HIV prevalence estimates were standardised for age, age and location (clinic for ANC-based estimates and individual-residence for population-based estimates), and age and clinic catchment by applying the respective prevalence estimates to samples of women adjusted to proportionally match ACDIS population level data on all women aged 15 to 49 as of 1^st ^January 2005. Women reported to the ACDIS during twice yearly fieldworker visits as having been pregnant (regardless of outcome) during the period 1^st ^July 2004 and 30^th ^June 2005, who were also eligible for population-based testing, were identified to assist comparative analyses.

Unrepresentative testing by HIV status was analysed by linking records (based on a unique identifier allocated to all participants) between the first (July 2003 to December 2004) and second (January to December, 2005) population-based HIV surveys. The proportion of women with a negative HIV test result in the first survey who also consented to test in 2005 was applied to all women with a first survey test result from whom consent was sought in 2005.

## Results

### Residency location and clinic catchment

**Figure 1:**
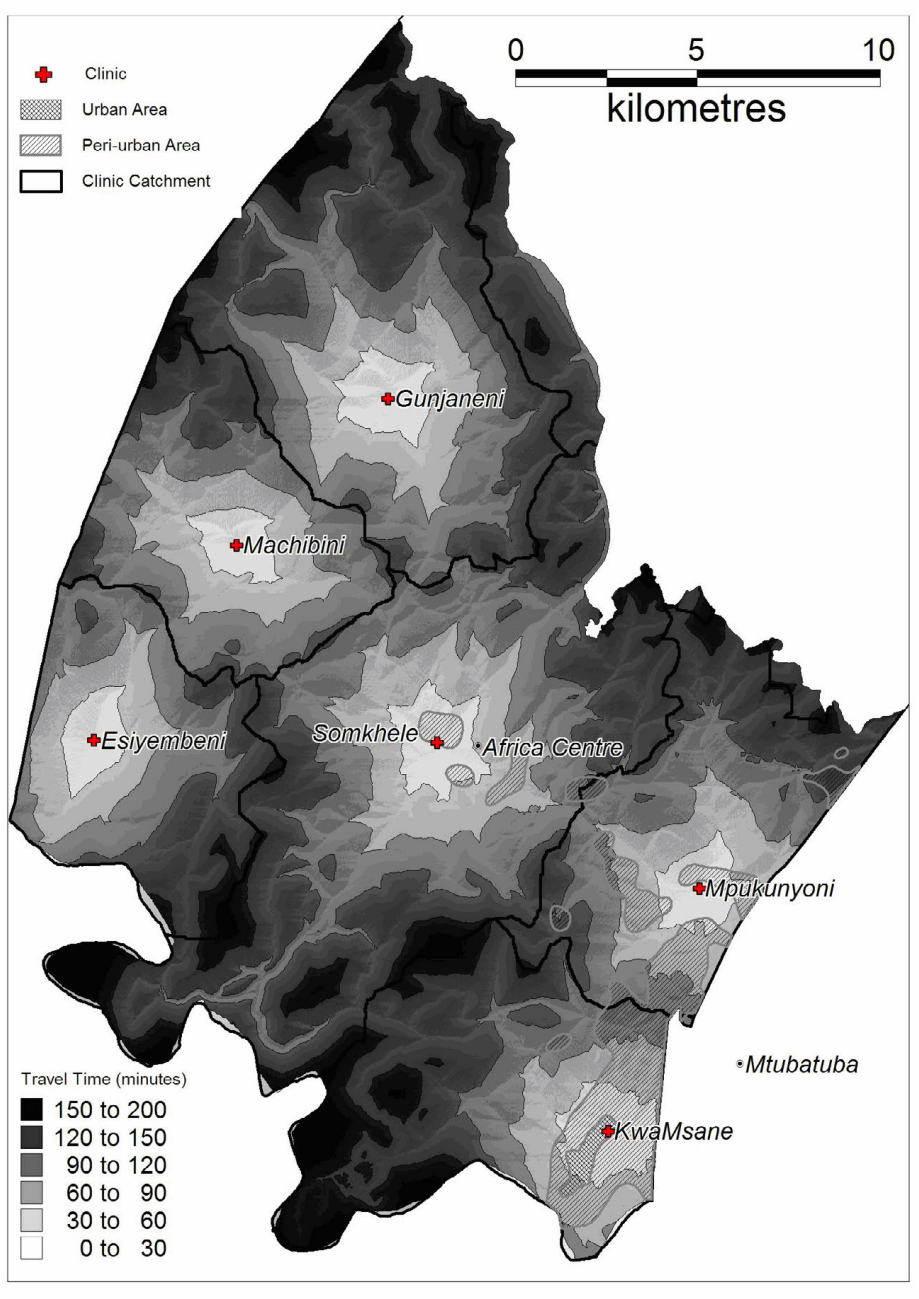
Estimated travel time to clinic and resulting catchments of the six government clinics within the surveillance area offering ANC by residency type.

Clinic attendance by residency type (where one or both is either predicted or reported) is presented for women eligible for testing in the population-based survey (Figure 1 and Table 1) as well as for women attending ANC first visits (Table 1). There was a 77% (2513/3281) agreement between reported antenatal clinic usage among women ever reporting a pregnancy (for whom information on both ANC clinic of attendance and residency is recorded) and usage as predicted by the clinic accessibility model. Compared to general clinic usage as reported across 23,000 homesteads within Hlabisa health sub-district [27], model predictions were 91% accurate.

**Table 1:** Women eligible for population-based HIV testing and women reported through ANC sentinel surveillance by residency location and clinic catchment

		**Clinic catchment type**							
				
	**Residency location**	**Peri-urban/Urban**		**Rural/peri-urban**		**Rural**		**Total**	
		
		**n**	**%**	**n**	**%**	**n**	**%**	**n**	**%**
**Women eligible for population-based testing ever reporting a pregnancy *(reported residency, reported clinic attendance)***	**Urban**	9	5.1	0	0.0	1	0.5	10	2.2
	**Peri-urban**	97	55.1	18	22.5	15	7.9	130	29.1
	**Rural**	70	39.8	62	77.5	174	91.6	306	68.6

**Total**		**176**	**100**	**80**	**100**	**190**	**100**	**446**	**100**

**All women eligible for population-based HIV testing *(reported residency, predicted clinic attendance)***	**Urban**	943	19.5	0	0.0	0	0.0	943	6.5
	**Peri-urban**	2748	56.9	1302	41.2	367	5.9	4417	30.5
	**Rural**^1^	1139	23.6	1859	58.8	5873	94.1	9116	63.0

**Total**		**4830**	**100**	**3161**	**100**	**6240**	**100**	**14476**	**100**

**Pregnant women eligible for population-based testing**^2,3 ^***(reported residency, predicted clinic attendance)***	**Urban**	52	11.1	0	0.0	0	0.0	52	3.6
	**Peri-urban**	279	59.7	136	40.6	43	6.9	458	31.7
	**Rural**	136	29.1	199	59.4	582	93.1	934	64.7

**Total**		**467**	**100**	**335**	**100**	**625**	**100**	**1444**	**100**

**ANC sentinel surveillance *(predicted residency, reported clinic attendance)***	**Urban**	116	19.5	0	0.0	0	0.0	116	10.5
	**Peri-urban**	339	56.9	72	41.2	23	6.6	434	39.0
	**Rural**	140	23.6	104	58.8	317	93.4	561	50.5

**Total**^4^		**595**	**100**	**176**	**100**	**340**	**100**	**1111**	**100**

1 Total includes 245 women not assigned to one of the six clinics within the ACDIS

2 Total includes 17 women not assigned to one of the six clinics within the ACDIS

3 Women reported to the ACDIS as pregnant between 01/07/04 and 30/06/05

4 Total excludes 35 women for whom there was no HIV test result (total ANC sample 1146)

### Population based HIV surveillance

Ten percent (1444/14476) of eligible women reported being pregnant between 1^st ^July 2004 and 30^th ^June 2005. Of the 14476 women eligible for HIV testing in 2005, 85.3% (12351) were contacted and asked to provide a blood sample. This proportion varied by age-group, parity and pregnancy status (all p < 0.001). Of those eligible, 36.6% (5293/14476) of women agreed to test, and of those contacted 42.9% (5293/12351) tested (Table 2). The proportion of women contacted who agreed to test varied by age-group, parity, residency location and clinic catchment (all p < 0.001). Pregnant women aged 15–19 years were most likely to consent to test (55.3%), whereas urban residents were least likely (12%). By age-group, the proportion of women consenting to test compared to those refusing significantly differed in all groups (p < 0.001) apart from the 20 to 24 year group (p = 0.111).

**Table 2:** Population-based HIV survey 2005: rates of testing consent and HIV prevalence estimates

**Women**	**Age group**	**Women eligible for inclusion in the population-based survey**	**Of women eligible for HIV testing those asked to consent to test**		**Of all women contacted those consenting to test**		**Of all women contacted those not consenting to test**		**Diagnosed with HIV infection**^3^		**95% CIs**		**Odds ratios**	**95% CIs**	
								
			**n**	**%**^1^	**n**	**%**^2^	**n**	**%**^2^	**n**	**%**					
**All**	**15–19**^5^	3669	3207	87.4	1658	51.7	1549	48.3	114	6.9	5.7	8.3	**-**	-	-
	**20–24**	2967	2307	77.8	1030	44.6	1277	55.4	294	28.7	26.0	31.6	**5.4**	4.3	6.8
	**25–29**	1842	1478	80.2	501	33.9	977	66.1	248	49.6	45.1	54.1	**13.2**	10.2	17.2
	**30–34**	1563	1327	84.9	470	35.4	857	64.6	220	47.0	42.4	51.6	**11.9**	9.2	15.5
	**35+**	4435	4032	90.9	1634	40.5	2398	59.5	451	27.7	25.6	30.0	**5.2**	4.1	6.4
	**Total**	**14476**	**12351**	**85.3**	**5293**	**42.9**	**7058**	**57.1**	**1327**	**25.2**	**24.0**	**26.4**	**-**	**-**	**-**

**Pregnant**^4^	**15–19**^5^	335	311	92.8	172	55.3	139	44.7	19	11.2	6.9	16.9	**-**	-	-
	**20–24**	475	427	89.9	200	46.8	227	53.2	52	26.1	20.2	32.8	**2.8**	1.6	5.0
	**25–29**	244	215	88.1	77	35.8	138	64.2	32	41.6	30.4	53.4	**5.7**	2.9	10.9
	**30–34**	183	169	92.3	64	37.9	105	62.1	21	32.8	21.6	45.7	**3.9**	1.9	7.9
	**35+**	207	197	95.2	95	48.2	102	51.8	20	21.3	13.5	30.9	**2.1**	1.1	4.3
	**Total**	**1444**	**1319**	**91.3**	**608**	**46.1**	**711**	**53.9**	**144**	**23.8**	**20.5**	**27.4**	**-**	**-**	**-**

**Parity (all women)**	**Nulliparous**^5^	5282	4324	81.9	1960	45.3	2364	54.7	232	11.9	10.5	13.4	-	-	-
	**Parity 1+**	9194	8027	87.3	3333	41.5	4694	58.5	1095	33.0	31.5	34.7	**3.7**	3.1	4.3

**Residency location (all women)**	**Rural**^5^	9116	7790	85.5	3560	45.7	4230	54.3	755	21.3	20.0	22.7	-	-	-
	**Peri-urban**	4417	3756	85.0	1636	43.6	2120	56.4	547	33.5	31.2	35.9	**1.9**	1.6	2.1
	**Urban**	943	805	85.4	97	12.0	708	88.0	25	26.0	17.6	36.0	**1.3**	0.8	2.1

1 Presented as a proportion of all women eligible for inclusion in the 2005 population-based survey

2 Presented as a proportion of all women contacted by HIV survey fieldworkers between January and December 2005

3 HIV test result not available for 27 women (8 nulliparous; 19 parity 1+;4 pregnant women) providing a blood sample

4 Reported as pregnant between 1st July 2004 and 30th June 2005

5 Baseline groups for odds ratios

A definitive HIV test result was available for 99.5% (5266/5293) of women testing. Amongst all women, HIV prevalence was 25.2% (95% CIs: 24.0%, 26.4%, median age 24 years), and in pregnant women 23.8% (95% CIs: 20.5%, 27.6%, n = 604, median age 21 years). Prevalence of HIV varied significantly by age-group amongst all women and pregnant women (both p < 0.001), (Table 2). Crude and age-standardised estimates for pregnant women were significantly lower than for women with a previous live birth who were not currently pregnant and significantly higher than for non-pregnant nulliparous women (both p < 0.001).

### Antenatal sentinel surveillance

An HIV test result was available for 96.9% (1111/1146) of women attending ANC first visits (median parity 1) and both a test result and date of birth available for 95.4% (1093/1146) of women (median age 23 years). Among the 1111 women for whom there was a test result, 12.7% (141) were reported as attending one of four rural-based clinics, 33.8% (375) one of two peri-urban-based clinics and 53.6% (595) the urban-based clinic (Table 1). Overall prevalence was 37.7% (414/1111), and was highest in the urban-based clinic with a predicted peri-urban/urban catchment, in women with a previous live birth, and amongst those aged 25 to 29 years (Table 3). Prevalence of HIV infection was shown to vary significantly by residency location (p = 0.032); clinic catchment (p = 0.028); parity and age (both p < 0.001). Standardising for age removed the significant difference in HIV prevalence estimates by residency location and clinic catchment (p = 0.084 and p = 0.057, respectively).

**Table 3:** ANC sentinel surveillance 2005: HIV prevalence estimates

		**Sample**^1 ^**(n)**	**Diagnosed with HIV infection**		**95% CIs**		**Odds ratio**	**95% CIs**	
								
			**n**	**%**					
**HIV Prevalence**^2^		1111	419	37.7	34.9	40.6	**-**	**-**	**-**

**Antenatal Clinic by location type**^2^	**Urban**^3^	595	243	40.8	36.9	44.9	-	-	-
	**Peri-urban**	375	135	36.0	31.1	41.1	**0.8**	0.6	1.1
	**Rural**	141	41	29.1	30.0	38.4	**0.6**	0.4	0.9

**Antenatal Clinic by Catchment type**^2^	**Peri-urban/urban**^3^	595	243	40.8	36.9	44.9	-	-	-
	**Rural/peri-urban**	176	68	38.6	31.4	46.3	**0.9**	0.7	1.3
	**Rural**	340	108	31.8	26.8	37.0	**0.7**	0.5	0.9

**Parity**^2^	**0**^3^	430	106	24.7	20.6	29.0	**-**	-	-
	**1**	324	150	46.3	40.8	51.9	**2.6**	1.9	3.6
	**2**	157	85	54.1	46.0	62.1	**3.6**	2.5	5.3
	**3+**	166	67	40.4	32.8	48.2	**2.1**	1.4	3.0
	**Not known**	34	11	32.4	17.4	50.5	**-**	-	-

**Age**	**15–19**^3^	251	49	19.5	14.8	25.0	**-**	-	-
	**20–24**	408	151	37.0	32.3	41.9	**2.4**	1.7	3.5
	**25–29**	210	114	54.3	47.3	61.2	**4.9**	3.2	7.4
	**30–34**	120	62	51.7	42.4	60.9	**4.4**	2.7	7.1
	**35+**	104	38	36.5	27.3	46.6	**2.4**	1.4	3.9
	**Not known**	18	5	27.8	9.7	53.5	**-**	-	-

1 35 women for whom there was no HIV test result removed from these analyses

2 Estimates includes 18 women for whom age was not reported

3 Baseline groups for odds ratios

### Comparing HIV prevalence estimates from the two surveillance systems

**Figure 2:**
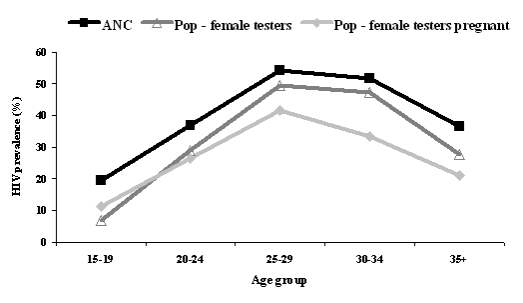
Population-based and ANC sentinel-surveillance age-specific HIV prevalence estimates.

Age-specific patterns of HIV prevalence in the ANC and population-based surveillance systems were similar (Figure 2). However, most crude population-based estimates were statistically lower (p < 0.05) than crude ANC-based estimates when disaggregated by pregnancy status, parity, location, and clinic catchment type (Table 4). Only among the 25–29 and 30–34 age-groups and the peri-urban location (residency or clinic) group did the two systems provide statistically similar estimates.

**Table 4:** Comparing population-based and ANC sentinel-surveillance HIV prevalence estimates

			**ANC based HIV prevalence estimates**^1^	**Population based HIV prevalence estimates**	**Ratio of ANC and population based estimates**^2^	**C hi^2 ^(p)**^3^
**All women**		**Crude**	**37.9**	**25.2**	**1.50**	**< 0.001**
		**15–19**	19.5	6.9	2.83	< 0.001
		**20–24**	37.0	28.7	1.29	0.002
		**25–29**	54.3	49.6	1.09	0.254
		**30–34**	51.7	47.0	1.10	0.362
		**35+**	3 6.5	27.7	1.32	0.053
		**Age standardised**	36.2	27.5	1.32	< 0.001
		**Age & location**^4^	31.7	27.5	1.15	0.005
		**Age & catchment**^5^	35.6	27.7	1.29	< 0.001

**Nulliparous**		**Crude**	**24.8**	**11.9**	**2.08**	**< 0.001**
		**Age standardised**	21.3	15.5	1.37	0.003
		**Age & location**^4^	20.1	15.9	1.26	0.037
		**Age & catchment**^5^	20.6	16.0	1.29	0.022

**Parity 1+**		**Crude**	**46.4**	**33.0**	**1.41**	**< 0.001**
		**Age standardised**	43.0	34.4	1.25	< 0.001
		**Age & location**^4^	37.0	34.1	1.09	0.153
		**Age & catchment**^5^	41.6	34.5	1.21	< 0.001

**Pregnant women**		**Crude**	**-**	**23.8**	**1.59**	**< 0.001**
		**Age standardised**	-	25.2	1.44	< 0.001
		**Age & location**^4^	-	25.3	1.25	0.006
		**Age & catchment**^5^	-	25.0	1.42	< 0.001

**Clinic location (ANC); residency location (population)**	**Urban**	**Crude**	**40.7**	**26.0**	**1.56**	**0.006**
		**Age standardised**	39.2	27.1	1.47	0.023
	**Peri-urban**	**Crude**	**36.5**	**33.5**	**1.09**	**0.277**
		**Age standardised**	36.8	36.5	1.01	0.930
	**Rural**	**Crude**	**29.2**	**21.3**	**1.37**	**0.028**
		**Age standardised**	28.5	23.2	1.22	0.151

**Projected clinic catchment (ANC); residence within projected clinic catchment (population)**	**Peri-urban/urban**	**Crude**	**40.7**	**30.5**	**1.33**	**< 0.001**
		**Age standardised**	38.2	33.5	1.14	0.039
	**Rural/peri-Urban**	**Crude**	**39.4**	**27.2**	**1.45**	**0.001**
		**Age standardised**	38.2	29.3	1.30	0.018
	**Rural**	**Crude**	**32.0**	**20.8**	**1.54**	**< 0.001**
		**Age standardised**	32.3	22.7	1.42	< 0.001

1 18 women excluded from the analyses with age not known

2 Ratio based on ANC totals when no direct comparable ANC group

3 Difference between two proportions (ANC-based HIV prevalence estimate compared to the population-based estimate)

4 Standardisation based on age and residency location (urban, rural and peri-urban) distribution within the ACDIS population

5 Standardisation based on age and predicted ANC clinic catchment (peri-urban/urban, peri-urban/rural and rural) distribution within the ACDIS population

The following adjustment factors would be necessary to adjust crude ANC-based estimates to match those provided by the population-based survey: 0.7 amongst all women; 0.6 amongst pregnant women; 0.5 amongst nulliparous women; 0.7 amongst women with a previous live birth. By primarily adjusting for an over-sampling of women aged 15–19 years in the population-based surveillance, and for an under-sampling of women aged 35+ years in ANC surveillance, age-standardisation reduced differences between the two sources of prevalence estimates, and removed the statistically significant difference in the rural location group (Table 4). Although age-standardisation increased the overall population-based estimate by 9.1% and decreased the ANC-based estimate by 4.2%, the difference between the two overall adjusted estimates remained significant. Age and clinic/residence location standardisation removed a statistically significant difference between the surveillance methods in women with a previous live birth (Table 4).

A test result from the first population-based survey was available for 28.8% (2030/7058) of women who refused to test in 2005. Women with a negative HIV test result from the first population-based survey were more likely to test in 2005 than those with a positive result (61.1% (2322/3801) compared to 52.6% (611/1162), p < 0.001). The figures for pregnant women were statistically similar to those for all women. Applying the consent rate of 61.1% to all women with a first survey test result from whom consent was sought in 2005 made little difference, with the adjusted estimate of 26.4% (1426/5392) remaining significantly lower than the overall ANC-based estimate, (both p < 0.001). Prevalence of HIV among women who tested during the first survey but who refused during the second survey was 1.3 times higher than prevalence among women who tested in both (27.1% [551/2030] compared to 20.8% [611/2933]).

## Discussion

The results of this paper show population-based estimates of HIV prevalence in women to be consistently lower than ANC-based estimates. Although there are several possible explanations for this difference (discussed below), one possible explanation that should first be considered is unrepresentative testing in the population-based survey

Within the population-based survey, women in the 25–29 and 30–34 age-groups presented both the highest HIV prevalence estimates and the lowest proportions agreeing to test. Women resident in the urban area were the overall group least likely to consent to test. Prevalence estimates among groups where the proportion of women contacted consenting to test is particularly low should be interpreted with caution. The proportions of women consenting to test are presented as of those contacted and not as of the full eligible population. Therefore, it may be likely that in terms of HIV status those contacted differ to those not contacted. Further analyses are necessary to explore this possible bias.

It is likely that within the ACDIS area, where provision of ANC clinics offering PMTCT services is comprehensive [29], many women will already be aware of their HIV status, and this may well influence their decision to agree to give a sample in population-based surveillance. Although testing consent in 2005 was lower amongst women with a previous HIV positive test result than amongst women with a previous negative test result, crudely adjusting for testing bias by HIV status had little effect. It should be noted that although test results are made available to participants [27] a low rate of uptake of test results [25] ensures a surveillance test result is no indication of an individual being aware of their HIV status.

A review of 20 national population-based surveys across sub-Saharan Africa suggests non-responders are likely to have a higher prevalence of HIV than responders and, by applying the most extreme scenario to account for such bias, an adjustment factor of 1.34 may be required [30]. Our analyses suggest prevalence among women testing in the first population-based survey but not the second was 1.3 times higher than that among women testing in both. However, it is unlikely that this figure is representative of all women refusing to test and therefore, it was not used to adjust the population-based estimate.

Although statistically similar, the overall population-based estimate for women of 25.2% (95% CIs: 24.0%, 26.4%) was lower than the estimate of 27.3% (95% CIs: 26.3%, 28.4%) from the first ACDIS population-based survey [24,31] and the estimate of 30.4% (95% CIs: 24.7%, 36.7%) amongst 15–49 year old women resident in 2005 in a province wide population-based survey of KwaZulu Natal [32].

The overall ANC-based estimate of 37.7% (95% CIs: 34.9%, 40.6%), although statistically similar, falls mid-way between estimates of 35.3% (95% CIs: 33.7, 36.9%) amongst pregnant women tested within the Hlabisa sub-District PMTCT programme January to June 2005 [29,33] and 39.1% (95% CIs: 36.8%, 41.4%) amongst 15–49 year old ANC clinic attendees in a province wide survey of KwaZulu Natal 2005 [34]. The estimate presented in this paper of 40.7% (95% CIs: 36.7%, 44.8%) amongst women attending the urban clinic is comparable to the estimate of 41.2% (95% CIs: 34.7%, 47.9%) for the same clinic in December 1998 [35].

### Comparative analyses of population-based and ANC-based estimates elsewhere

Modern contraceptive use in South Africa is the highest in Sub-Saharan Africa and fertility the lowest [21]. In contrast with the results presented here, regional and national studies in sub-Saharan Africa, including Tanzania [36-38], Uganda [39], Zambia [18-20] and Cameroon [2], found ANC-based estimates to be lower than estimates amongst women in the population. Across a range of sub-Saharan African countries ANC-based estimates have been shown to be on average 28% lower than population-based estimates for women [1]. Comparing estimates at the national level with other national or regional estimates can be problematic due to bias as a result of site selection. ANC-based results presented here and those from other regional surveys conducted elsewhere in sub-Saharan Africa show higher prevalence estimates among women attending urban-based clinics than clinics based elsewhere [8].

A study based on regional estimates in three sub-Saharan African countries suggested that to convert ANC-based estimates amongst primagravida to all childless women in general populations with high contraceptive use (20%+) an adjustment factor of 0.6 would be necessary [1]. Amongst multigravida it was suggested an adjustment factor of 1.1 would be necessary to represent all mothers [1]. In the ACDIS population, an adjustment factor of 0.5 was necessary to match the ANC-based estimate amongst nulliparous women to that provided by the population-based survey, whereas amongst women with a previous live birth the figure was 0.7.

### Additional explanations for differences in prevalence estimates

#### a) Age, contraceptive-use and fertility

The largest differences between the two surveillance systems were in women aged 15–19 years and nulliparous women. That ANC-based estimates were so much higher than population-based estimates in these two groups probably reflects self-selection bias amongst ANC attendees with regards age of sexual debut and non-contraceptive use. Although women aged 25–34 years have the lowest consent rates for population-based testing and highest HIV prevalence, adjusting for unrepresentative testing by age only removes a significant difference within the rural location group.

In areas with high contraceptive use ANC-based estimates would be expected to exceed population-based estimates among women whereas, where fertility rates are reduced due to prevalence of HIV infection the opposite is true [1]. Although use of modern contraceptives is high and fertility has declined within the ACDIS [21], and although HIV prevalence amongst currently pregnant women in the population was estimated to be significantly lower than that amongst women with a previous live birth, it was not possible to separate out the influences of contraceptive use or HIV related sub-fertility.

#### b) Unrepresentative selection of clinics in ANC sentinel surveillance

A study of bias in ANC-based surveillance data suggested that an over-estimation of HIV prevalence due to an over representation of urban-based clinics could be mitigated if urban and peri-urban based clinics were shown to be attracting large numbers of women from rural areas [8]. The results presented here show the urban-based clinic to contribute over half of all ANC-based HIV test results and suggest over 70% of women attending the urban or peri-urban clinics are resident in an area type other than that where the clinic is located. It should be noted, that the relatively small size of the urban and peri-urban areas [28,40] may facilitate this process. Furthermore, the urban-based clinic is located at the southeast extremity of the study area and may well attract individuals from urban areas lying outside of the study area. It was for this reason ANC-based estimates were standardised by age. Since ANC surveillance did not collect residency location it was not possible to assess whether ANC clinic attendance outside of residency location was associated with HIV status.

Despite supportive evidence for the accuracy of the predictive model for clinic catchments presented both previously [28,40] and here, and despite predicted high levels of clinic attendance outside of residency location, standardising estimates by age and clinic catchment did little to reduce differences between the two sources of prevalence estimates. However, standardising estimates by age and location did reduce differences. Age and location standardisation may well provide the most robust estimates for rural and peri-urban areas (standardised estimates for urban location [area with highest ANC-based prevalence estimate, highest out-of-area clinic attendance and lowest population-based consent rate] remained significantly different).

#### c) Unrepresentative reporting and under-reporting of pregnancies

In an area with high ANC coverage [29] and where pregnant women in the population present the highest overall rate of testing consent and similar rates of testing consent by HIV status than amongst all women, it is difficult to explain why crude and standardised population-based estimates presented for pregnant women and by parity differ so greatly to ANC-based estimates. It is possible that pregnant women in the two populations are not fully comparable.

A study in Hlabisa sub-district showed that 91% of homesteads normally utilise the most accessible primary health clinic [28]. However, it is likely that a proportion of women attending the urban clinic reside outside of the surveillance area. Under-reporting of pregnancies ending in early term HIV-related pregnancy loss in ACDIS would result in pregnant women living with HIV/AIDS, who may have already attended an ANC first visit, not being identified within the population-based survey. Not only are further analyses of how pregnancies are reported to demographic systems warranted, so are analyses of how multiple methods of HIV testing in an area influence decisions to test within population-based surveillance. A greater understanding of how high levels of contraceptive use and possible HIV associated sub-fertility effect HIV prevalence estimates is also required.

## Conclusion

The findings of this study suggest that where ANC coverage is high, population-based HIV surveillance systems under-estimate HIV prevalence due to unrepresentative testing by HIV status that results in unrepresentative testing by age and residence. The results also suggest that despite evidence of large numbers of women from rural areas (where HIV prevalence tends to be lower) attending urban and peri-urban clinics, in an area with high contraceptive use and low fertility, resulting in selection bias due to age of sexual debut, ANC sentinel surveillance over-estimates prevalence. Understanding how to appropriately adjust ANC sentinel surveillance estimates to represent HIV prevalence in general populations is important since ANC clinics continue to be a relatively cheap and timely source of data.

The findings of this study highlight the possible biases inherent to both surveillance systems and suggest that attention should be paid to unrepresentative HIV testing, particularly by age and residency location. Analysing population-based HIV prevalence estimates, and comparing them to ANC-based estimates, also highlights the importance of not assuming population-based estimates equate to a gold standard.

## Competing interests

The author(s) declare that they have no competing interests.

## Authors' contributions

All authors read and approved the final manuscript prior to submission.

BDR carried out data-cleaning of ANC dataset as well as the majority of the analyses and writing.

JBF coordinated the 2005 population-based HIV survey and 2005 ANC sentinel surveillance, and conducted data cleaning of population-based surveillance data.

VH assisted data analyses.

FT assisted data analyses.

CH assisted data analyses.

TB assisted data analyses.

KH assisted with the overall approach of the analyses and writing.

TW designed the ANC sentinel surveillance and assisted with the structure of the writing.

MLN assisted with the overall approach of the analyses and writing. All authors read and approved the final manuscript.

## Pre-publication history

The pre-publication history for this paper can be accessed here:



## Supplementary Material

Additional file 1 **Additional information**. A word document that presents key messages, statement, ethics, keywords and abbreviations should any of these be needed. Click here for file
