# Supplementary material for: Population and antenatal-based HIV prevalence estimates in a high contracepting female population in rural South Africa
Source: BMC Public Health. 2007 Jul 18;7:160. doi: 10.1186/1471-2458-7-160 (PMC1948890; doi:10.1186/1471-2458-7-160)
Supplement: Additional file 1 — Additional information. A word document that presents key messages, statement, ethics, keywords and abbreviations should any of these be needed. [file 1471-2458-7-160-S1.doc]

**Key messages**

1. Overall population-based HIV prevalence estimates amongst all women (25.2%) and pregnant women (23.8%) from the Africa Centre Demographic Information System, KwaZulu Natal, South Africa were significantly lower than the overall ANC-based estimate (37.9%) from the same area.
2. Women in the 25-29 and 30-34 age-groups in the population-based survey presented both the highest HIV prevalence estimates and the lowest proportions agreeing to test. The results also suggest lower consent rates amongst women who know or suspect themselves to be HIV positive.
3. To mitigate concerns of urban clinic bias in ANC-based HIV prevalence estimates large numbers of women from rural areas, where HIV prevalence tends to be lower, were estimated to attend urban and peri-urban clinics offering ANC services.
4. Standardising for age and location (clinic and individual residency type) may well provide the most robust HIV prevalence estimates (particularly for rural and peri-urban areas).
5. The results suggest that where ANC coverage and contraceptive use is widespread and fertility is low, population-based surveillance systems under-estimate HIV prevalence due to unrepresentative testing by age, residence and also probably by HIV status, and that despite evidence of large numbers of women from rural areas attending urban and peri-urban clinics, ANC sentinel surveillance over-estimates prevalence due to selection bias in terms of age of sexual debut and contraceptive use.

**Statement**

The Africa Centre population-based HIV survey was established in 2003 as an annual cross-sectional survey of all women aged 15 to 49 years and all men aged 15 to 54 years either resident within the Africa Centre Demographic Surveillance Area or included within a sample of individuals resident elsewhere but remaining as a member of a household within the area. Both the population-based and ANC sentinel surveillance database are held at the Africa Centre for Health & Population Studies and strict attention to confidentiality is maintained at every stage of data collection, analysis and storage.

**Ethical Approval**

The University of KwaZulu Natal Ethics Committee approved the following

applications: “A proposal for the creation of a longitudinal population-based HIV

research platform and non-communicable disease surveillance in the Africa Centre Demographic Information System, Hlabisa, South Africa. AJ Herbst, Africa Centre” (E029/03) 3^rd^ June 2003 and “A socio-demographic platform for population-based reproductive health research in a rural district of KwaZulu-Natal” (E009/00) 6^th^ September 2000. The application for “Africa Centre ANC surveillance in government clinics of part of Hlabisa sub-District” was approved by the KwaZulu Natal Provincial AIDS Action Unit 26^th^ May 2003.

**Keywords**

HIV, prevalence, bias, population, antenatal-care, South Africa

**Abbreviations used**

Africa Centre Demographic Information System (ACDIS); antenatal care (ANC); geographical information system (GIS)
